# Characterization of Synaptically Connected Nuclei in a Potential Sensorimotor Feedback Pathway in the Zebra Finch Song System

**DOI:** 10.1371/journal.pone.0032178

**Published:** 2012-02-22

**Authors:** Shayna M. Williams, Alexis Nast, Melissa J. Coleman

**Affiliations:** 1 Claremont McKenna College, Claremont, California, United States of America; 2 Scripps College, Claremont, California, United States of America; 3 W. M. Keck Science Department of Claremont McKenna College, Pitzer College, and Scripps College, Claremont, California, United States of America; University of Maryland, United States of America

## Abstract

Birdsong is a learned behavior that is controlled by a group of identified nuclei, known collectively as the song system. The cortical nucleus HVC (used as a proper name) is a focal point of many investigations as it is necessary for song production, song learning, and receives selective auditory information. HVC receives input from several sources including the cortical area MMAN (medial magnocellular nucleus of the nidopallium). The MMAN to HVC connection is particularly interesting as it provides potential sensorimotor feedback to HVC. To begin to understand the role of this connection, we investigated the physiological relation between MMAN and HVC activity with simultaneous multiunit extracellular recordings from these two nuclei in urethane anesthetized zebra finches. As previously reported, we found similar timing in spontaneous bursts of activity in MMAN and HVC. Like HVC, MMAN responds to auditory playback of the bird's own song (BOS), but had little response to reversed BOS or conspecific song. Stimulation of MMAN resulted in evoked activity in HVC, indicating functional excitation from MMAN to HVC. However, inactivation of MMAN resulted in no consistent change in auditory responses in HVC. Taken together, these results indicate that MMAN provides functional excitatory input to HVC but does not provide significant auditory input to HVC in anesthetized animals. We hypothesize that MMAN may play a role in motor reinforcement or coordination, or may provide modulatory input to the song system about the internal state of the animal as it receives input from the hypothalamus.

## Introduction

Songbirds are used as a model system to understand the neural basis for learned motor behaviors, particularly vocalizations. Learned vocalizations require the integration of auditory signals with appropriate motor output to shape the target sound. In addition, maintenance of song requires feedback about the ongoing motor pattern, and the ability to modulate the motor pattern. In zebra finches, (*Taeniopygia guttata*) song is a male-specific behavior that is controlled by a set of identified nuclei, known collectively as the song system. The song system can be divided into two main pathways: the anterior forebrain pathway (AFP) and the vocal motor pathway. The AFP is part of a basal ganglia forebrain loop that is primarily involved in song learning and plasticity, while the vocal motor pathway is required for song production. This report begins to analyze the role of a thalamo-cortical pathway in maintenance of this complex learned behavior by first characterizing the impact of this pathway on a key vocal motor nucleus, HVC.

HVC is a cortical region critical for song learning and production that has both pre-motor neurons and neurons that project to the AFP [1], [2], [3], [4], [5], and it is thought to contain the pattern generating circuit for song [6], [7], [8], [9]. HVC neurons also receive auditory input that is highly selective for the bird's own song (BOS) [1], [10], [11], [12], [13], [14]. Therefore, HVC is a potential site of sensorimotor integration and is a focus of many studies aimed at understanding auditory motor integration, particularly for learned behavior. The four known inputs to HVC are the caudal mesopallium (CM), interfacial nucleus of the nidopallium (NIf), nucleus uvaeformis (Uva), and medial magnocellular nucleus of the nidopallium (MMAN). CM and NIf provide the major auditory input to HVC [15], [16], [17]. Uva projects directly to HVC as well as indirectly via NIf [18], [19], appears to provide modulatory input to HVC [18], and is also important for interhemispheric coordination of HVC activity [20]. The role of MMAN's input to HVC is less clear.

**Figure 1:**
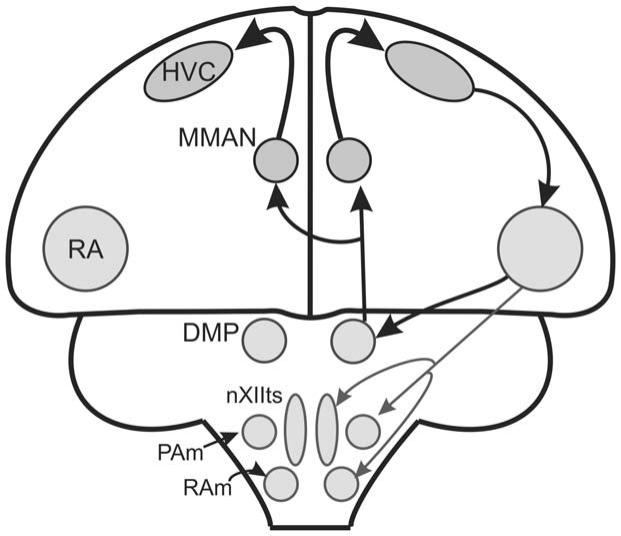
Schematic of the feedback loop to HVC through MMAN. For clarity, anatomical connections are only shown for the right hemisphere. HVC projects to the robust nucleus of the archipallium (RA), which projects to the (DMP). DMP projects bilaterally to MMAN. RA also projects to premotor nuclei that control the syrinx and respiration (PAm and RAm). Abbrev: DMP, dorsomedial nucleus of the posterior thalamus; MMAN, medial magnocellular nucleus of the nidopallium; nXIIts, tracheosyringeal part of the hypoglossal nucleus; PAm, paraambigualis; RAm, retroambigualis.

MMAN is particularly interesting as it not only projects to HVC, but it also forms a potential sensorimotor feedback loop via the robust nucleus of the arcopallium (RA) and the dorsomedial nucleus of the posterior thalamus (DMP) (Figure 1) [21], [22]. This feedback loop is interesting for at least three reasons. First, in many experience-dependent pathways there is feedback to regions involved in motor production – the most studied of which are thalamo-cortical feedback loops in vertebrates [23], [24]. The loop involving MMAN and HVC represents a thalamo-cortical feedback loop that may be important for song learning and production. Although not tested here directly, MMAN may provide motor feedback to the song system. Second, MMAN anatomically receives input from the hypothalamus, via DMP, presenting the possibility of input to the song system about the internal state of the animal [25]. This input may influence when a bird sings, which could be important for survival and reproductive success. Third, DMP projects bilaterally to both the ipsilateral and contralateral MMAN, making this loop one of only two known bilateral pathways in the song system. The only other known bilateral pathway is through Uva [19]. Because song production requires bilateral control of the vocal organ [26], [27], interhemispheric coordination is paramount in neural control of song.

While the function of MMAN remains unclear, several lines of evidence suggest that it plays an important role in song learning and maintenance. First, directed singing increases expression of the immediate early gene, early growth response-1 (egr-1; or ZENK, a zinc-finger-containing transcriptional regulator), indicating that MMAN is active during motor production of song [27]. Second, bilateral MMAN lesions in juveniles result in the development of highly abnormal, short, and relatively unstereotyped song, indicating that MMAN is necessary for normal song learning [17]. While bilateral MMAN lesions in adults with stereotyped song do not result in major song abnormalities as seen in juveniles, the lesions do cause a consistent increase in song variability – especially at the beginning of song production [21]. These effects of MMAN lesions on song learning and maintenance could be related to the disruption of a sensorimotor-feedback loop involving MMAN and HVC. Third, MMAN also displays auditory activity and responds to auditory playback of the BOS [28]. Although almost all of the auditory activity in HVC originates from NIf and CM there could be other auditory inputs to HVC as preliminary data suggest that bilateral lesions of these two areas do not result in song degradation, as seen with deafening [28]. Taken together, these data suggest that MMAN is involved in sensorimotor feedback, this feedback is necessary for song learning and maintenance, and MMAN may provide sensorimotor feedback information to HVC to modulate ongoing motor output during song production. This idea is supported by a study showing that MMAN activity does not show greater response to the first syllable of bird's own song playback (as is seen in HVC) and that song-evoked bursts of activity in MMAN can last greater than 100 ms after onset of song [28].

To further understand the role of MMAN's input to HVC, we characterized the auditory responses in both areas and confirm and extend previous reports that MMAN responds selectively to auditory information and that functionally excites HVC. Because MMAN responds selectively to BOS, we also tested whether MMAN provides auditory input into HVC by measuring HVC's auditory response when MMAN was inactivated. We had two alternative hypotheses: 1) MMAN provides auditory input into HVC, and thereby represents a fourth auditory input into HVC that is required for song learning, or 2) MMAN does not provide auditory input into HVC and therefore may have another role such as to provide motor feedback or additional sensory information to HVC necessary for modulation of song production.

Some of these data have appeared in abstract form [29].

## Materials and Methods

### Subjects

A total of 36 adult (>90 days post hatch) male zebra finches (*Taeniopygia guttata*) were used for this study. All procedures performed in this study were done so in accordance with a protocol approved by the Institutional Animal Care and Use Committee at the W.M. Keck Science Department of Claremont McKenna College, Pitzer College and Scripps College. All efforts were made to minimize suffering. All birds used in this study were obtained from the colony in the W.M. Keck Science Department or from a local supplier. All birds were provided food and water *ad libitum* and were on a 14∶10 hour day:night light cycle.

### Stimuli

Before each experiment, the song of each male bird was recorded by placing the bird in a sound-attenuation chamber (Eckel Industries, Cambridge, MA) with a female bird. Songs were recorded using Sound Analysis Pro [30]. Songs were filtered (high-pass 300 Hz, low-pass 8000 Hz) and edited using Goldwave (Goldwave Inc., St. John's, Newfoundland, CAN). Edited songs included 2–3 motifs, the largest repeatable unit of a song, for the bird's own song (BOS), the BOS in reverse (REV), and conspecific (CON) song. All songs were presented at ∼70 dB (SPL), measured with a sound level meter (rms, A-weighted, RadioShack).

### Surgery

Prior to each experiment, recorded birds were anesthetized with a total of 90–100 µL of 20% urethane, administered in 3 injections of 30–40 µL in the pectoral muscle over the course of 1 hour. Two hours after the last injection, lidocaine (2%, Hospira Inc., Lake Forrest, IL) was injected under the scalp, and the scalp was dissected along the midline. The approximate x-y location of MMAN was marked on the surface of the skull and a head post was mounted to the anterior part of the skull with dental cement (Coltene/Whaledent Inc., Cuyahoga Falls OH) and cyanoacrylate (Krazy Glue™). Once the cement hardened, the bird was placed on a heating pad (FHC, Bowdoin, ME) on an air table (TMC, Peabody, MA) surrounded by sound foam attached to the interior wall of a faraday cage. The mounted head post immobilized the bird's head, and the body temperature was held constant (37°C). The head angle of the bird was set 40° relative to horizontal. A speaker was placed approximately 35 cm in front of and facing the bird.

### Electrophysiology and song presentations

Multiunit extracellular recordings were made with carbon fiber electrodes (Kation Scientific, Minneapolis, MN). Small craniotomies were made in the skull above the approximate locations of MMAN and HVC, and electrodes were lowered into the brain using micromanipulators (Siskiyou, Grants Pass, OR; Newport). All recordings were amplified (A-M Systems, Sequim, WA), filtered (300 Hz highpass, 5 kHz lowpass), digitized at 20 kHz (Micro1401, CED, Cambridge, England) and collected using Spike 2 software (CED). For HVC, the final electrode position was approximately 2.4 mm lateral of the bifurcation of the midsagittal sinus and 200 to 500 µm ventral to the dorsal surface of the brain. For MMAN, the final electrode position was 5.2 mm anterior and 0.5 mm lateral of the bifurcation of the midsagittal sinus and 1.8 to 2.0 mm ventral to the dorsal surface of the brain. Both nuclei were identified by their individual characteristic firing pattern, correlated spontaneous activity [31] and auditory responses (see results). All recordings were from the ipsilateral MMAN and HVC.

Spontaneous and song-evoked activity was recorded in MMAN and HVC simultaneously. For each recording, 20 to 40 repetitions of each song type (BOS, REV and CON) were interleaved with a 7±2 second inter-stimulus interval. After each recording session, electrolytic lesions (+10 µA for 5 seconds) were made at the MMAN recordings site to enable histological confirmation of the recording location (see below).

To characterize the synaptic latency between MMAN and HVC, MMAN was stimulated (A-M Systems Model 2100) while an extracellular recording was made in HVC. All stimuli were single pulses 0.3 ms in duration and 10–50 µA in amplitude. The threshold for eliciting a response in HVC was 10–20 µA.

For the inactivation of MMAN, MMAN and HVC were first located by using carbon fiber electrodes to record from MMAN and HVC, then the carbon fiber electrode in MMAN was then replaced with a glass electrode filled with 250 mM GABA (Sigma-Aldrich) in 1 M NaCl. Song-evoked activity was then collected for 10 to 40 repetitions of each song before inactivation (pre), during inactivation (GABA), and 5–10 minutes after GABA application (post). GABA was puffed (30–50 ms at 16–20 psi) out of the recording pipette with a picospritzer (Toohey Co., Fairfield, NJ). For some experiments, a small quantity of rhodamine dye (∼0.5–1%) was mixed with the GABA and puffed into brain to mark the location and spread of the inactivation. In experiments where dye was not used, the location of the injection site was marked by making an electrolytic lesion (+10 µA for 5 seconds). The inactivation site was later identified histologically.

### Histology

**Figure 2:**
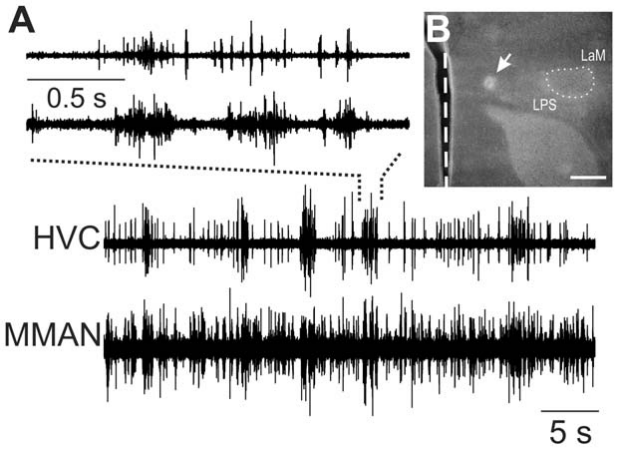
Spontaneous bursts of action potentials are correlated in HVC and MMAN. A. Simultaneously recorded spontaneous activity in the ipsilateral MMAN and HVC. The top two traces are an expansion of the recording in the bottom two traces (dotted line). B. Coronal section showing the lesion (arrow) of the recording site for experiment in A. Note the location of the lesion medial to the lateral magnocellular nucleus of the anterior nidopallium (LMAN; dotted line) and in between the mesopallial lamina (LaM) and lamina pallio-subpallialis (LPS). Scale bar, 500 µm. The dashed vertical line denotes the midline. Dorsal is upward.

After each experiment, the bird was euthanized with a lethal dose of Nembutal (0.05 cc, 50 mg/mL) and perfused transcardially with 0.9% saline followed by 4% paraformaldehyde (in 0.025 M NaPO_4_ buffer). The brain was then removed from the skull and stored in 4% paraformaldehyde until histological processing. Brains were cryoprotected in 30% sucrose in 4% paraformaldehyde overnight and then sectioned coronally on a freezing microtome (Microm) into 70 µm sections. Lesion sites were identified after the slices were stained with cresyl violet. Digital images of the rhodamine labeling were superimposed on images of the same section viewed under combined darkfield and fluorescent illumination (CorelDraw). MMAN is located medial to LMAN and between the mesopallial lamina (LaM) and lamina pallio-subpallialis (LPS) [25]. LMAN can be readily identified with darkfield illumination or from the cresyl violet staining as the size of its cell bodies is much larger than those in the surrounding tissue (see Figure 2B). Identification of MMAN in cresyl violet-stained sections is very difficult so a recording was considered in MMAN if the lesion or fluorescent marker was located in between the two laminae and medial to LMAN.

### Data analysis

To quantify the auditory response to BOS, REV, and CON in MMAN and HVC, the response strength and z-scores were calculated using a MATLAB script (written by E.S. Fortune). The response strength is calculated as the difference between the mean multiunit firing rate (spikes/second) during song playback stimulus and the mean firing rate during a baseline pre-stimulus period (1.5–2.5 s) of the same duration. We use the term ‘spikes’ to refer to any event over a user-defined threshold. Because of the high degree of variability in response strength, response strengths were also normalized and expressed as z-scores. The z-score is calculated as the difference between the firing rate during the stimulus and the baseline firing rate divided by the standard deviation of the difference:
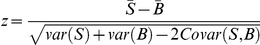
where 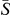 is the mean firing rate during song playback, 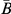 is the mean baseline firing rate, and the standard deviation is calculated by taking the square root of the variance of 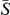 plus the variance of 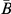 minus the covariance of 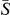 and 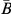 [17].

The selectivity of the response in HVC and MMAN to one stimulus compared to another was measured using the d′ metric. This metric provides a statistical measure for the discriminability between two stimuli [32]. The d′ value was calculated using a MATLAB script (written by E.S. Fortune and edited by J. McGrady Achiro) using the following equation:
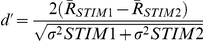
where 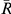 is the mean response strength to the stimulus (STIM), and σ^2^ is its variance. For our analyses, the selectivity for BOS (STIM1) was compared with REV and CON (STIM2). A d′ of 0.5 was used as the criterion for deeming a response selective [33].

## Results

### Spontaneous activity in MMAN and HVC

Like other canonical nuclei of the song control circuit, MMAN showed characteristic spontaneous bursts of activity that were correlated with spontaneous bursts in HVC [17] (Figure 2). Overall, MMAN displayed more background activity than HVC (HVC, 13.8±2.0 spikes/s; MMAN, 20.0±3.0 spikes/s; paired t-test, p<0.01). To confirm our recording site, in most experiments we lesioned the recording site (Figure 2B). MMAN is not easily identifiable by Nissl stain, unlike most nuclei in the song system. It is located medial to the lateral nucleus of the anterior nidopallium (LMAN) and between two fiber tracts, the lamina mesopallialis (LaM) and lamina pallio-subpallialis (LPS) [21]. Although we could not absolutely confirm the location of the recording site in every experiment, we determined that we were recording from MMAN based on its electrophysiological properties, which included correlated spontaneous bursts of activity with those in the ipsilateral HVC and auditory selectivity for the BOS. Our electrode tracks were always just lateral to the midsagittal sinus and thus medial to LMAN [22] (see Figure 2).

### Auditory response in MMAN and HVC

**Figure 3:**
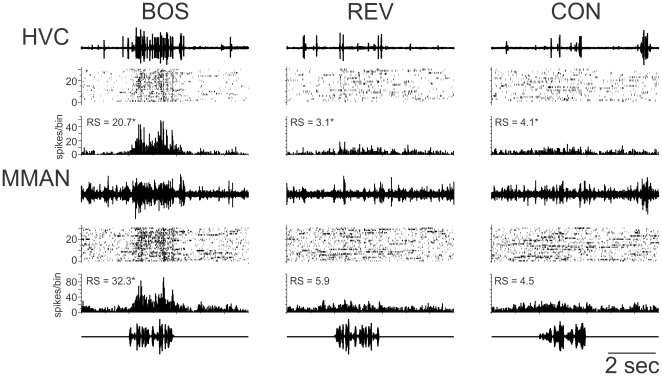
Auditory-evoked action potential activity in MMAN and HVC. Simultaneous multiunit activity from ipsilateral MMAN and HVC in response to playback of the bird's own song (BOS), the BOS in reverse (REV), and conspecific (CON) song. For both HVC and MMAN, top row, raw data for a single playback of each song; middle row, raster plot of activity to thirty iterations of each song; bottom row, peri-stimulus time histogram (PSTH) of the cumulative response to each song playback. Bin size = 25 ms. The response strength (RS) for each response is given, * indicates a RS that was significantly greater than 0 (one-tailed t-test; p<0.05). For MMAN response to REV, p = 0.06; CON, p = 0.08.

HVC responds selectively to playback of BOS over other stimuli, including REV and CON [1], [10], [11], [12], [13], [34]. A previous report showed auditory responses in MMAN that were also selective for BOS over REV [31]. To further characterize the auditory responses in MMAN and HVC, we made simultaneous extracellular recordings from ipsilateral HVC and MMAN and presented auditory stimuli (Figure 3; n = 17 in 16 birds). Playback of BOS, REV, and CON elicited auditory responses in MMAN similar to those recorded simultaneously in HVC (Figure 3). MMAN showed a significant response over baseline to playback of BOS and CON, but not REV (one-tailed t-test; Table 1), whereas HVC showed a significant response to BOS, REV and CON (Table 1).

**Table 1:** Response strength (units/s) and z-scores for simultaneous recordings in HVC and MMAN to auditory playback (N = 17 from 16 birds).

	BOS	p-value	REV	p-value	CON	p-value
HVC RS	23.07±2.29	<0.001	3.16±0.86	0.052	3.55±0.98	<0.05
MMAN RS	11.51±2.29	<0.001	0.69±0.86	0.431	2.32±0.98	<0.05
HVC z-score	1.83±0.42	<0.001	0.28±0.125	<0.05	0.41±0.14	<0.01
MMAN z-score	0.88±0.18	<0.001	−0.16±0.27	0.551	0.195±0.08	<0.05

All values are mean ± SEM.

**Figure 4:**
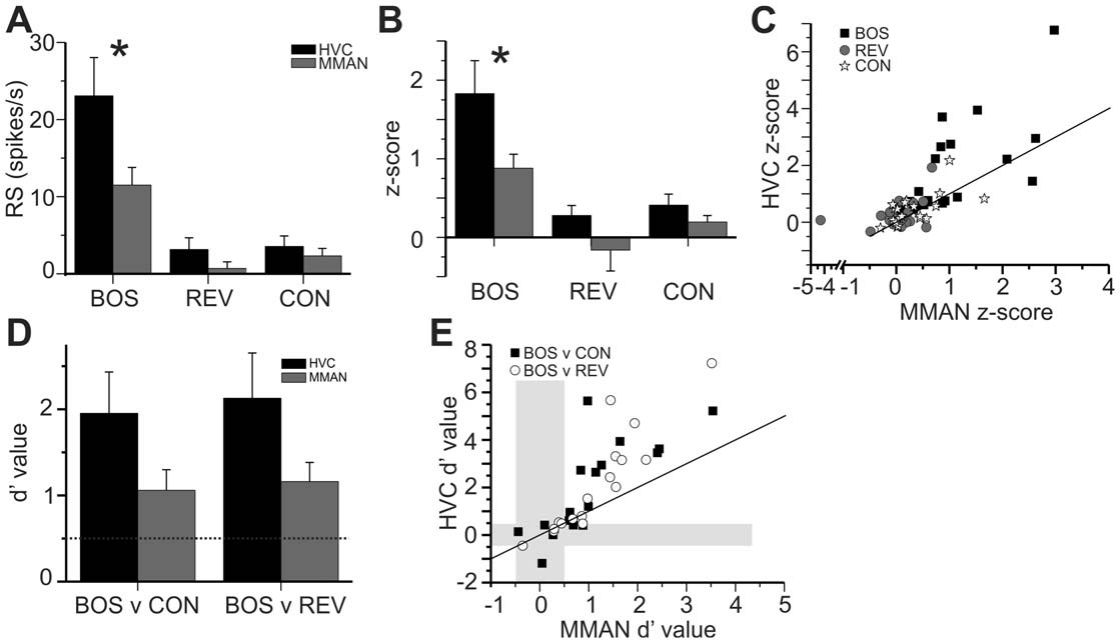
Comparison of auditory responses and song selectivity in simultaneously recorded multiunit activity in MMAN and HVC. A. Comparison of the response strength in HVC and MMAN. HVC had a significantly larger RS to BOS than did MMAN (*; paired t-test, p<0.05). The response to REV and CON in HVC and MMAN were not significantly different (REV, p = 0.17; CON, p = 0.47). B. Z-score for auditory-evoked activity in HVC and MMAN. HVC had a significantly higher z-score to BOS than did MMAN (*; paired t-test, BOS, p<0.01). The response in HVC and MMAN to REV and CON were not significantly different (REV, p = 0.20; CON, p = 0.15). C. Comparison of z-scores from simultaneous recorded activity in HVC and mMAN. Black diagonal line is the unity line. D. Both HVC and MMAN were selective for BOS vs REV and BOS vs CON. Selectivity was defined as d′>0.5 (dashed horizontal line). E. HVC was more selective for BOS versus CON than simultaneously recorded MMAN, as many points lie above the unity line than expected at random (p<0.05). The points for BOS versus CON did not lie significantly above the unity line than expected at random (p>0.05). The grey bars demark non-significant d′ values for MMAN and HVC; i.e., −0.5>d′>0.5.

Both MMAN and HVC had a significantly greater response to BOS than to REV or CON (z-scores used for calculation: one-way ANOVA, p<0.05, F = 10.6 for HVC; F = 7.76 for MMAN; Tukey HSD p<0.05). MMAN responded significantly less to BOS than did HVC (z-score values, paired t-test, p<0.01), but did not have a different response to REV and CON than HVC (z-score values, paired t-test, REV; p = 0.15, CON; p = 0.20). A direct comparison of simultaneously recorded auditory responses in ipsilateral HVC and MMAN to auditory stimuli revealed that, within a recording, HVC responded more to BOS than MMAN (Figure 4C; points lie above the unity line; p<0.01). In addition, there was little difference between the response to REV and CON in simultaneous recordings from MMAN and HVC as those points were clustered around the unity line (Figure 4C; REV, p = 0.23; CON, p = 0.12). For this analysis, significance was determined by resampling procedures in R to determine the likelihood that the observed number of points would lay above the unity line at random. Briefly, 10000 resamples from the original data were carried by randomizing the HVC values (with replacement), re-pairing them with the MMAN values, and re-calculating the percentage of points that lay above the line.

The selectivity of HVC and MMAN for BOS over other auditory stimuli can also be measured using the d′ value, a statistical measure of discriminability between two stimuli [35]. A significant preference for one stimulus over another is defined as −0.5>d′>0.5. Both HVC and MMAN showed significant preference for BOS over REV and CON (Figure 4D; BOS v CON: HVC, 1.95±0.5; MMAN, 1.06±0.2; BOS v REV: HVC, 2.13±0.5; MMAN, 1.16±0.22). In addition, HVC had significantly greater d′ values for BOS versus CON and for BOS versus REV than did MMAN (paired two-tailed t-test, p<0.05) (Figure 4D). Comparing d′ values from simultaneous recordings in MMAN and HVC (Figure 4E) we found that more points were significantly above the unity line for BOS v CON (p<0.05), supporting the idea that HVC was more selective than simultaneously recorded MMAN. However, for BOS over REV, the points were not significantly above the unity line, suggesting that HVC was not more selective for BOS over REV (p>0.05; resampling analysis as for z-score values). If the non-significant responses in MMAN (points in the grey) were removed for both BOS v REV and BOS v CON, then the remaining values did lie significantly above the unity line (p<0.05). Thus, if MMAN is selective for BOS over REV or CON, then the response in HVC is statistically greater. Thus, like HVC, MMAN neurons are more selective to auditory playback of BOS over other auditory stimuli. However, HVC neurons are more selective for BOS over other stimuli than MMAN.

### Stimulation

**Figure 5:**
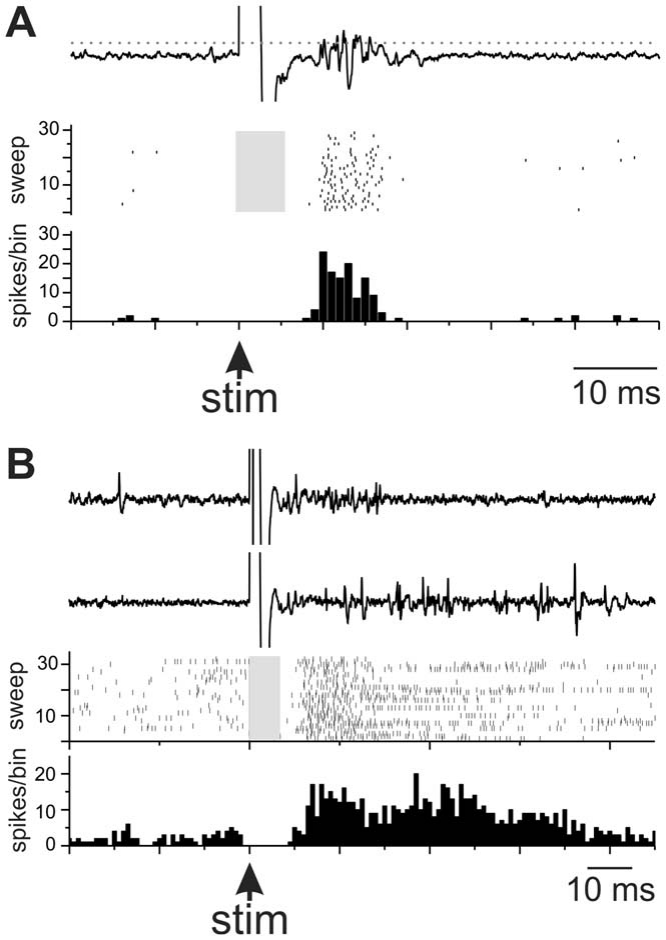
Stimulation of MMAN functionally excites the ipsilateral HVC. A. Example of the short latency response from MMAN stimulation to HVC response. Top trace is an exemplar of the raw HVC response to MMAN stimulation (large artifact). The dotted grey line denotes the spike threshold set by the user. Middle trace, raster plot of responses to thirty stimulus pulses in MMAN. Grey bar indicates time in which stimulus artifacts were removed from the plot. Bottom trace, PSTH of HVC response to MMAN stimulation. B. Longer latency response in HVC to MMAN stimulation. Top two traces, raw exemples of two HVC responses to stimulation of MMAN. Middle trace, raster plot to of HVC response to 30 MMAN stimulations. Grey bar indicates time in which stimulus artifacts were removed from the plot. Bottom trace, PSTH of the cumulative response in HVC to MMAN stimulation. For both A and B, bin size = 1 ms. A and B are from two different birds.

To examine the functional synaptic input from MMAN to HVC, we stimulated MMAN while recording extracellularly in the ipsilateral HVC (Figure 5). Stimulation in MMAN resulted in a complex excitatory response in HVC (n = 3). In one case there was a clear and consistent excitatory response in HVC with a delay between 10–17 ms (Figure 5A). In two cases, the MMAN stimulation resulted in a very long-lasting response in HVC. The example shown had a response between 9–70 ms (Figure 5B). The initial response (first peak) appeared to be consistent with the time of the response shown in Figure 5A. The second phase of the response could be due to recurrent activation of the feedback loop through HVC. Stimulation outside of MMAN did not result in a consistent latency response in HVC (n = 3; data not shown). These data suggest that MMAN provides functional excitatory input to HVC, with a synaptic delay of 10–17 ms, consistent with a previous report [25].

### Inactivation of MMAN

**Figure 6:**
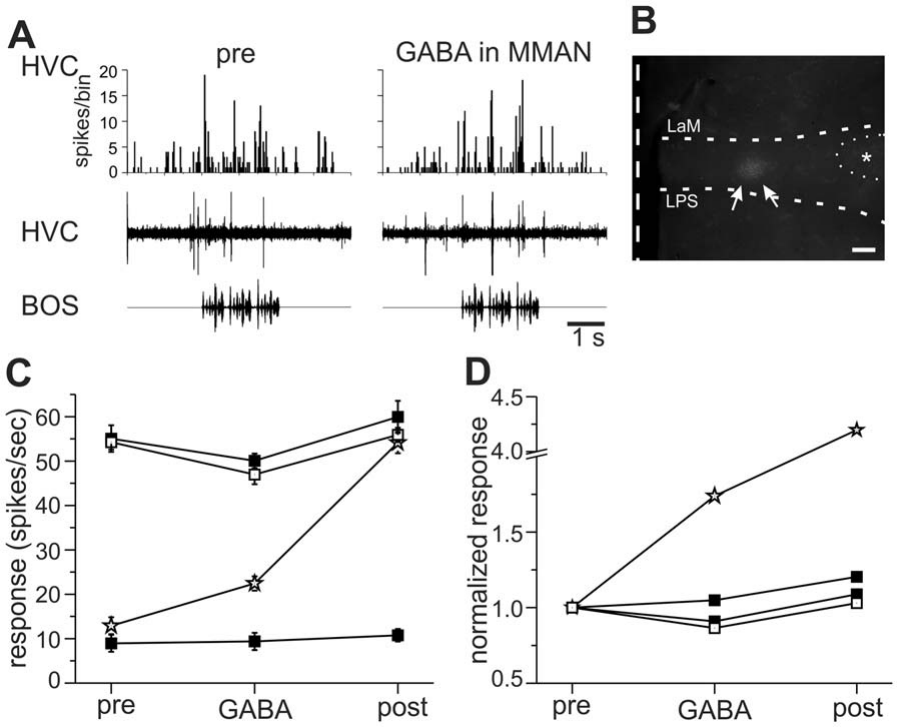
Inactivation of MMAN resulted in little change in HVC auditory responses. A. Example of auditory evoked activity in MMAN and HVC before (left) and with (right) GABA application to MMAN. Top trace, PSTH of HVC response to ten iterations of BOS playback. Bin size, 25 ms. Middle trace, single, raw example of multiunit activity in HVC. Bottom trace, sonogram of BOS. B. Location of GABA application in MMAN approximated by rhodamine labeling (arrows). LMAN is outlined to the right of the dye (dotted semicircle, *). Scale bar, 200 µm. The dashed vertical line denotes the midline. Dorsal is upward. C. Average response of HVC to BOS presentation before (pre), during (GABA) and after (post) BOS presentation. In two experiments (filled squares) GABA application to MMAN did not produce a significant change in HVC response. In one experiment (open squares) there was a significant decrease in the HVC response to BOS during GABA application compared to pre and post GABA application (ANOVA, p<0.5, Tukey post-hoc). In one bird (open stars) the response to BOS increased throughout the duration of the experiment (pre, GABA, and post were all significantly different than each other; p<0.5, ANOVA, Tukey post-hoc). D. Normalized responses of data shown in C.

As MMAN responds to auditory stimulation and provides functional excitatory synaptic input to HVC, it is possible that MMAN also provides sensory feedback to HVC and contributes to the auditory response in HVC. GABA_A_ receptors have been localized in MMAN [36], so to test this idea we inactivated MMAN with GABA while recording auditory evoked activity in HVC (n = 4 in 3 birds). The effect of GABA in MMAN on auditory responses in HVC was calculated using the activity (response) during auditory stimuli (Figure 6), as the response strength was more variable, presumably due to a large variability in baseline activity. GABA inactivation of MMAN had no significant effect on ipsilateral HVC auditory activity in 2 of 4 experiments (Figure 6; one-way ANOVA, p>0.05). In one experiment there was a continual increase in auditory response, even after GABA had washed out of MMAN (stars in Figure 6C&D). This response was unusual, and each condition (pre, GABA, post) was significantly different from the others (Tukey post-hoc p<0.05). One other experiment showed a significant decrease in response to GABA inactivation of MMAN compared to pre and post (open squares in Figure 6C&D; ANOVA, p<0.05, Tukey post-hoc test). Pre and post were not different from each other (p>0.05, Tukey post-hoc test). The site of GABA injection was histologically confirmed in all cases (see Figure 6B for an example). In summary, inactivation of MMAN with GABA had little reliable effect on auditory response in HVC. These data suggest that MMAN is not a significant source of auditory input to HVC and its auditory activity may be the result of input through the feedback pathway.

**Figure 7:**
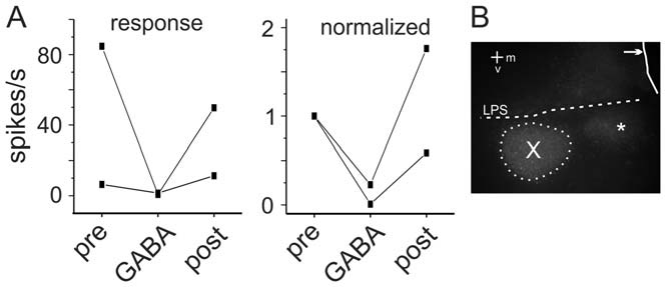
GABA inactivation of the area ventral to MMAN resulted in a reduction in auditory responses in HVC. A, Left, HVC response to ten iterations of BOS before, during, and after GABA application. Right, normalized response to BOS. B. Location of the GABA injection (*). Dye was located ventral to LPS and medial to Area X (outlined by dotted line). The midline is indicated by the line (arrow). Scale bar, 200 µm. M, medial; V, ventral.

In two experiments in which MMAN was missed, GABA application resulted in a dramatic decrease in auditory responses in HVC (Figure 7A; one-way ANOVA, p>0.05, Tukey post-hoc). The HVC auditory response was significantly smaller during GABA application compared to pre- and post- application (Tukey post-hoc, p<0.05). Histological analysis showed that, in these cases, GABA was injected ventral to MMAN (Figure 7). In both cases, there was little to no action potential activity in HVC during auditory playback when this area was inactivated. Furthermore, inactivation of this area not only decreased auditory response in HVC but also greatly reduced all activity in HVC (data not shown). The identity of this area is not known, but may be the medial part of Area X [37]. The rhodamine dextrane that was co-applied with the GABA showed retrogradely labeled cells in Area X (Figure 7B) indicating that Area X neurons project to this area.

## Discussion

To gain a better understanding of the influence of MMAN on HVC activity we recorded simultaneously from these two areas. We found similar auditory responses in HVC and MMAN, although HVC was more selective for BOS over other songs than MMAN. This could be due to increased signal to noise as a consequence of lower spontaneous activity in HVC compared to MMAN. In addition, we found that stimulation of MMAN functionally excited HVC. We found that MMAN inactivation had little effect on auditory-evoked responses in HVC, indicating that MMAN does not provide significant auditory input to HVC, in anesthetized birds.

A direct comparison of the timing between ipsilateral MMAN and HVC auditory-evoked activity shows a complex interaction between MMAN and HVC activity where sometimes HVC activity leads that in MMAN and sometimes MMAN activity leads that in HVC [31]. Using cross-correlation analysis of multiunit activity in MMAN and HVC, Seti and Okanoya (2008) found that sometimes HVC led MMAN activity by 2–25 ms and sometimes MMAN led HVC activity by 10–25 ms. The delays between MMAN and HVC auditory activity are consistent with the electrical stimulus-evoked activity in HVC, although we typically saw a longer delay in responses. A previous report showed that electrical stimulation of HVC resulted in a 4–20 ms delay in neural response in MMAN [25]. The complex timing is mostly likely due to the feedback loop from the cortex through the thalamus back to the cortex. Single unit recordings from MMAN and HVC may help resolve the more ambiguous timing between the two nuclei, however this is unlikely to fully resolve this issue as the same phenomenon has been shown in lateral MAN (LMAN) and HVC using intracellular recordings from both sites [38]. Another way to potentially resolve the ambiguous timing is to repeat the experiments presented here after lesioning RA to functionally remove the feedback.

Although MMAN is selective for auditory playback of the BOS and stimulation of MMAN excites HVC, inactivation of MMAN has no consistent effect on auditory responses in HVC. This is in contrast to what is seen with inactivation of the two main known auditory inputs to HVC, NIf and CM, which results in a significant loss of auditory activity in HVC [15], [17]. The inconsistent effect of MMAN inactivation on HVC activity could be due to several factors, including an inconsistent volume of injected GABA and GABA injections that extended beyond the bounds of MMAN. We found that GABA application slightly ventral to MMAN resulted in a profound loss of auditory activity in HVC. The proximate location of this dorsal site to MMAN makes precise inactivation of MMAN even more difficult and could account for the small decrease in HVC auditory activity in one of the inacativation experiments. Further experiments are needed to more fully characterize the identity and influence of the unknown area on auditory activity in HVC. The small and inconsistent influence of MMAN on HVC auditory activity suggests MMAN plays another, perhaps modulatory role on HVC activity, or could provide input about other sensory (e.g. proprioceptive) information to HVC which could be important for modulation of song. One intriguing possibility is that MMAN provides input to the song system about the internal state of the animal, via its indirect input from the lateral hypothalamus [25]. This may be critical for regulating song production, and possibly song frequency, by integrating information about sexual maturity, the time of day, or other information regarding the internal state of the animal.

It is possible that the influence of MMAN on HVC auditory activity is dampened by the anesthesia, although the anesthetic used here usually enhances auditory responses in the canonical song system and auditory responses are greatly reduced in awake birds [39]. Future chronic recordings from awake, behaving finches will be needed to more fully determine the effects of MMAN on auditory and motor activity in HVC.

### Contributions of MMAN to bilateral coordination

It has been proposed that the feedback loop to HVC through MMAN may work to coordinate bilateral HVC activity because it allows motor information from one RA to reach the contralateral HVC via DMP and MMAN [20], [22]. This loop through DMP is one of only two known feedback loops with bilateral projections in the song system [19]. The other pathway projects from RA to PAm (paraambigualis, see Figure 1) or to DM (dorsomedial nucleus of the inter-collicular region, not shown), then from each of these to Uva (not shown), and back to HVC [20]. It has been shown that while bilateral lesions in Uva disrupt song production, unilateral lesions in Uva only disrupt song temporarily [20]. Interestingly, unilateral Uva lesions permanently disrupt song if MMAN is also impaired [40]. Bilateral lesions of MMAN in adult finches result in an increase in song variability at the beginning of a song, which is consistent with MMAN's role in coordinating activity between the two hemispheres. Taken together, these data suggest that MMAN is an important component of hemispheric coordination in the vocal motor pathway. Several reports suggest that the pattern generating circuit for song production is located in HVC [5], [7], [8]. In addition, activity in both hemispheres is highly coordinated [41]. Bilateral activation of both HVCs by MMAN may provide a way to provide consistent feedback that can coordinate bilateral activity in HVC, similar to activity that can reset activity in pattern-generating circuits.

### A novel auditory input to HVC?

Although inactivation of MMAN did not significantly alter auditory responses in HVC, we found, surprisingly, that inactivation of the area ventral to MMAN resulted in a dramatic reduction of spontaneous and auditory activity in HVC. The identity of this area is unknown, although it may be the medial part of Area X. Consistent with this result, Kubikova et al. (2007) showed lesions in medial Area X resulted in lower ZENK expression in the ipsilateral HVC than the contralateral HVC [37], while MMAN lesions did not result in differences in ZENK expression in ipsi- versus contralateral HVC. Future work will further characterize this area and its anatomic connectivity.

## Acknowledgments

We would like to thank Dr. T. Nick and B. Perlmutter for reading earlier versions of the manuscript.
